# Yellow Fever Vaccination in an Individual with Confirmed Egg Protein Allergy: A Case Report

**DOI:** 10.3390/vaccines14070606

**Published:** 2026-07-10

**Authors:** Nelly Otte, Amir Maleki Kahaki, Gerda Wurpts, Thomas Kraus, Julia Krabbe

**Affiliations:** 1 Institute of Occupational, Social and Environmental Medicine, Medical Faculty, University Hospital RWTH Aachen, 52074 Aachen, Germany; 2 Institute for Prevention and Occupational Medicine of the German Social Accident Insurance, Institute of the Ruhr University Bochum (IPA), Medical Faculty, Ruhr University Bochum, 44789 Bochum, Germany; 3 Aachen Comprehensive Allergy Center (ACAC), University Hospital RWTH Aachen, 52074 Aachen, Germany; 4 Department of Dermatology and Allergology, Medical Faculty, University Hospital RWTH Aachen, 52074 Aachen, Germany

**Keywords:** yellow fever vaccine, egg allergy, Stamaril^®^, fractional dose vaccination, occupational immunization, seroconversion, live-attenuated vaccine, travel medicine

## Abstract

Background: Yellow fever vaccination is recommended for travelers visiting endemic regions in South America and Africa. The live-attenuated vaccine (Stamaril^®^, Sanofi Pasteur) is produced in embryonated chicken eggs and contains residual ovalbumin, posing a challenge for individuals with egg protein allergy. Case Presentation: We report the case of a 35-year-old male disaster relief worker with confirmed egg protein allergy and moderate sensitization but no history of anaphylaxis. Under normal circumstances, a statement confirming that vaccination is not possible would be sufficient for entry. However, due to his work-related duties and the relevant guidelines from his employer, there was an urgent need for vaccination despite his egg protein allergy. A comprehensive allergy work-up including skin prick testing, serum-specific IgE, and oral provocation testing revealed a moderate allergy without systemic reactions. Intervention: Following informed consent and risk assessment, a fractional dose (0.1 mL) of the reconstituted yellow fever vaccine was administered under close monitoring. The patient developed a localized dermal reaction without systemic symptoms. Post-vaccination neutralization testing confirmed seroconversion indicating protective immunity. Discussion: Although yellow fever vaccination is considered contraindicated in individuals with severe egg protein allergy, the literature supports its cautious use in selected cases. Fractional dosing has emerged as an effective dose-sparing strategy. Studies show that even in egg protein-allergic individuals, adverse events are rare. Conclusions: In this case, a fractional yellow fever vaccination was safe and an effective option for an egg-allergic individual in a high-risk occupational setting, supporting a personalized approach based on clinical risk assessment and current evidence.

## 1. Introduction

Yellow fever is a viral hemorrhagic disease caused by the yellow fever virus, transmitted by *Aedes* and *Haemagogus* mosquito species in endemic tropical areas of South America and sub-Saharan Africa [1]. No specific antiviral treatment is available for yellow fever and management remains primarily supportive. The case fatality rate of severe yellow fever is approximately 20–50%, particularly among unvaccinated individuals [2].

To prevent transmission and outbreaks, proof of yellow fever vaccination is required for international travelers to and from endemic regions, as per International Health Regulations (IHR) [3]. The vaccine is generally well tolerated. Common adverse reactions include mild fever, headache, myalgia and local injection site reactions [1]. Rare but serious adverse events, such as yellow fever vaccine-associated viscerotropic disease and neurologic complications, occur with increased risk in individuals over 60 years of age or with thymic disorders [4]. Due to these risks, careful screening evaluation is required prior to immunization in vulnerable groups.

Yellow fever vaccines are produced in embryonated chicken eggs, resulting in residual ovalbumin content. Consequently, individuals with egg protein allergy have traditionally been considered at increased risk of immediate hypersensitivity reactions, including anaphylaxis [5].

The yellow fever vaccine (Stamaril^®^—Sanofi Pasteur) is a live-attenuated virus cultured in embryonated chicken eggs. As such, egg protein allergy is considered a contraindication for vaccination [1]. Nevertheless, certain occupational scenarios, e.g., humanitarian aid, disaster relief, and health service missions, can require urgent immunization even in patients with known allergies.

A single subcutaneous dose of 0.5 mL of yellow fever vaccine confers seroconversion in 95–100% of healthy adults within 10–30 days and is typically sufficient for long-term, potentially lifelong immunity. In individuals with HIV, in pregnant individuals, and in children under the age of two, booster doses may still be necessary, as a lower proportion of these groups maintain seroprotective antibody levels ten years or more after initial vaccination [6].

Due to global vaccine shortages and outbreak demands, fractional dosing of the yellow fever vaccine has emerged as a viable dose-sparing strategy. Clinical trials and field studies have demonstrated that administration of one-fifth of the standard dose (0.1 mL) induces neutralizing antibodies in over 95% of adult recipients, with sustained protective titers for at least 12 months [7]. In a randomized trial, four dose levels of the 17DD vaccine were tested, and even the lowest dose (≈0.1 mL) elicited protective responses without increased adverse effects [8].

We report the case of a patient with a confirmed egg protein allergy who, due to professional obligations in high-risk regions, underwent fractional-dose yellow fever vaccination under monitored conditions to minimize exposure to residual egg proteins.

## 2. Materials and Methods

### 2.1. Patient

A 35-year-old male disaster relief worker presented to the interdisciplinary outpatient clinic of the Aachen Comprehensive Allergy Center (ACAC), Germany, with a request for yellow fever vaccination. He worked as a global disaster relief professional and was on short-notice stand-by for deployment to tropical regions, including endemic countries in Africa and South America.

The patient had a known allergy to chicken egg protein since childhood, with clinical symptoms of perioral tingling and lip swelling after egg consumption, but no history of anaphylaxis. His medical history also included a controlled allergic asthma.

### 2.2. Skin Prick Testing

Skin prick testing was performed with histamine and saline as positive and negative controls. Raw and cooked chicken egg whites were taken from fresh eggs provided by the patient and skin prick-to-prick tests (SPPT) were performed. Wheal diameters were measured after 15 min; reactions ≥ 3 mm above the negative control were considered positive.

### 2.3. Serum-Specific IgE Testing

Serum-specific IgE antibodies against chicken egg white, egg yolk, and conalbumin were determined using the ImmunoCAP assay. Results were reported in kUA/L and classified according to the corresponding CAP classes. Total IgE was measured in serum and reported in kU/L (kilounits of allergen-specific IgE per liter).

### 2.4. Oral Provocation Test

Oral provocation with egg white was performed under supervision using graded doses up to half a chicken egg white.

### 2.5. Vaccination with Stamaril^®^

Vaccination with Stamaril^®^ (Sanofi Pasteur, Lyon, France) was administered subcutaneously into the upper arm a dose of 0.1 mL, followed by observation for three hours.

### 2.6. Serologic Titers Post-Vaccination

Serological titers were determined post-vaccination by detecting neutralizing antibodies using the plaque-reduction neutralization test (PRNT).

## 3. Results

Due to the contraindication of yellow fever vaccination in egg protein-allergic individuals, a comprehensive allergological work-up was initiated; see Table 1.

Despite confirmation of sensitization, particularly to raw egg components, the patient expressed a strong and persistent wish to proceed with immunization, citing occupational necessity and global deployment obligations.

Given the absence of a history of anaphylaxis and only mild reaction during provocation testing, we consulted the current literature, which described successful immunization with fractional doses (e.g., 0.1 mL) of yellow fever vaccine under close observation [7,8].

Following extensive counseling and informed consent, the patient received a fractional subcutaneous dose of 0.1 mL Stamaril^®^, representing one-fifth of the standard dose (0.5 mL). The procedure was conducted in a controlled clinical setting with anaphylaxis emergency equipment on stand-by.

Within 30 min of administration, the patient developed a localized dermal reaction: an erythematous area of approximately 30 mm in diameter and a small wheal at the injection site. No urticaria or systemic symptoms, e.g., bronchospasm or hypotension, were observed. The patient was monitored for two hours and discharged uneventfully.

To assess seroconversion, a neutralization test for yellow fever antibodies was performed 11 weeks post-vaccination. The titers were 105 and 119, indicating a successful immune response and presumed protective immunity.

## 4. Discussion

Yellow fever vaccines are produced in embryonated chicken eggs, resulting in residual ovalbumin content. Consequently, individuals with egg protein allergy have traditionally been considered at increased risk of immediate hypersensitivity reactions, including anaphylaxis [5]. Based on the Vaccine Adverse Event Reporting System, the Centers for Disease Control and Prevention determined an incidence rate of 14.6 anaphylaxis cases per 1,000,000 doses [9].

The described comprehensive diagnostic approach was chosen to thoroughly determine if a conventional full-dose vaccination could be performed. This was the first vaccination with egg-protein-containing vaccines for the patient. Non-egg-protein-containing vaccinations were administered without any complications or allergic reactions. Thus, the medical team was optimistic at first since the patient did not report any anaphylaxis in his lifespan. However, after the occurrence of gastrointestinal symptoms after ingestion of cooked egg white, in which most conalbumin would have been denaturized, sensitization against egg white and conalbumin was determined, and abstention from yellow fever vaccination was advised. A medical certificate confirming that vaccination was contraindicated was considered sufficient for travel to areas where yellow fever is endemic. Patients are advised to take extra precautions to protect themselves from mosquito bites to prevent the disease. However, due to his professional obligations and the guidelines established by his employer, vaccination was a mandatory requirement for entry, despite his protein allergy. The patient was determined to work in those areas and therefore requested vaccination despite his egg protein allergy.

The WHO lists egg protein allergy as a precaution, but not an absolute contraindication, provided appropriate precautions are taken [1]. Current yellow fever vaccination protocols do not specifically distinguish between pediatric and adult patients with egg protein allergy [10]. In patients with a history of anaphylaxis to egg protein, vaccination is typically avoided unless the risk of exposure is substantial. However, in those with milder forms of egg protein allergy, the vaccine may be administered under controlled conditions, including allergy work-up, skin testing, and in-clinic observation with emergency stand-by [11]. Children and adults with egg protein allergy exhibit largely similar safety profiles following yellow fever vaccination, though most data come from pediatric cohorts.

In a large study of 171 egg protein-allergic children (24% with prior anaphylaxis), all tolerated yellow fever vaccination without adverse reactions, regardless of skin test results [12]. This included those who received the vaccine in either a single or divided dose. Other studies have also reported vaccination without anaphylaxis in children with egg protein allergies [13].

In a large Brazilian pediatric cohort of 435 children with a history or suspicion of egg protein allergy, 95.2% tolerated the vaccine and only one case of possible anaphylaxis occurred, with skin testing proving unreliable as a predictor [14].

In the presented case, the patient had moderate sensitization to egg proteins, no history of anaphylaxis, and only mild gastrointestinal symptoms during oral provocation. These findings, combined with occupational necessity, justified consideration of vaccination under enhanced safety measures.

The WHO’s Strategic Advisory Group of Experts (SAGE) has recognized fractional dosing as an effective emergency tool, especially in situations where vaccine supplies are extremely limited, e.g., to stretch the vaccine, although it is not yet formally licensed for routine use [15]. While most data pertain to immunocompetent adults, its use in children, the elderly, or immunocompromised individuals remains under investigation [3].

Finally, a stepwise approach was chosen: a 0.1 mL dose was administered with the intent that if no symptoms occurred, the rest of the 0.5 mL could be administered right after. However, since a localized reaction without systemic symptoms was observed, it was decided not to administer the remaining dose. In consultation with the patient, it was agreed that an antibody titer test should be performed first, and that a repeat fractional vaccination would only be considered if the titer was found to be too low. In consultation with the Robert Koch Institute’s analytical laboratory, antibody testing was performed first. Based on other studies [15,16,17] and the laboratory’s reference values, seropositivity was assumed to be present between 1:50 and 1:10 sample dilution. Since a sufficient titer was detected, adequate protection was confirmed. Furthermore, antibody levels similar to full-dose vaccinations were achieved. In a study in children, more than half of the included children showed a titer lower than 1:100 sample dilution 1 year after vaccination [15]. In adults, higher titers were observed in some patients [17,18]. Some individuals with lower baseline immunity could benefit from a second dose as booster [19].

So, in this rare case, a fractional vaccination despite egg protein allergy was conducted although the patient was not a resident in an endemic region and could have easily avoided exposure in daily life. This could be of interest as an example for clinical practice in cases where the recommended approach for patients traveling to endemic areas cannot be applied and the benefits of vaccination outweigh the risk of anaphylaxis.

## 5. Conclusions

Yellow fever vaccination remains the cornerstone of preventive strategy in endemic regions. While egg protein allergy represents a challenge due to residual protein content, carefully selected patients may be safely immunized under controlled conditions. Fractional dosing not only offers a dose-sparing solution but may also reduce the allergenic burden and risk profile in sensitized individuals. This case illustrates how personalized medicine, grounded in allergy diagnostics and supported by international evidence on fractional immunization, can enable safe and effective vaccination in high-risk but essential occupational settings.

## Figures and Tables

**Table 1:** Allergy work-up prior to vaccination.

Assay	Potential Allergen	Result
Skin Prick Testing		Wheal Size (mm)
	Cooked Egg White	2
	Raw Egg White	7
	Positive Control (Histamine)	6
	Negative Control (0.9% NaCl)	no wheal
Serological Testing		Specific IgE Titer (kUA/L) ^1^
	Egg White	0.88 ^2^
	Egg Yolk	2.20 ^3^
	Conalbumin	0.43 ^4^
Oral Provocation Test		Symptoms
	Increasing doses at 60 min intervals (cumulative maximum ½ egg white)	Mild gastrointestinal symptoms (bloating, discomfort), no systemic or respiratory reactions.

^1^ Total IgE level 47.1 kU/L. ^2,3^ CAP class 2, moderate sensitization. ^4^ CAP class 1, low sensitization.

## Data Availability

As a case report about a single case no comprehensive data were created or analyzed in this study. Data sharing is not applicable to this article.

## Abbreviations

The following abbreviations are used in this manuscript:

ACAC	Aachen Comprehensive Allergy Center
CAP	Carrier-Polymer-System Test
IHR	International Health Regulations
PRNT	Plaque-Reduction Neutralization Test
SAGE	Strategic Advisory Group of Experts
SPPT	Skin Prick-to-Prick Test

## References

[1] Yellow Fever. https://www.afro.who.int/health-topics/yellow-fever.

[2] Colebunders R., Mariage J.L., Coche J.C., Pirenne B., Kempinaire S., Hantson P., Van Gompel A., Niedrig M., Van Esbroeck M., Bailey R. (2002). A Belgian traveler who acquired yellow fever in the Gambia. Clin. Infect. Dis..

[3] International Health Regulations 2005. https://iris.who.int/bitstream/handle/10665/246107/9789241580496-eng.pdf.

[4] Lindsey N.P., Schroeder B.A., Miller E.R., Braun M.M., Hinckley A.F., Marano N., Slade B.A., Barnett E.D., Brunette G.W., Horan K. (2008). Adverse event reports following yellow fever vaccination. Vaccine.

[5] Kelso J.M., Mootrey G.T., Tsai T.F. (1999). Anaphylaxis from yellow fever vaccine. J. Allergy Clin. Immunol..

[6] Schnyder J.L., De Jong H.K., Bache B.E., Schaumburg F., Grobusch M.P. (2024). Long-term immunity following yellow fever vaccination: A systematic review and meta-analysis. Lancet Glob. Health.

[7] Roukens A.H.E., Visser L.G. (2019). Fractional-dose yellow fever vaccination: An expert review. J. Travel Med..

[8] Martins R.M., Maia M.D.L.S., Farias R.H.G., Camacho L.A.B., Freire M.S., Galler R., Yamamura A.M.Y., Almeida L.F.C., Lima S.M.B., Nogueira R.M.R. (2013). 17DD yellow fever vaccine. Hum. Vaccin Immunother..

[9] McClenathan B.M., Taylor J.N., Housel L.A., Ryan M. (2024). Incidence of anaphylaxis to YF-VAX^®^ yellow fever vaccination: A retrospective evaluation of vaccine adverse event reports 1999–2018. J. Travel Med..

[10] CDC (2025). Yellow Book.

[11] Abrams E.M., Zafack J.G., Jensen C., Bettinger J.A., Top K.A., Hildebrand K.J., Tunis M.C. (2024). Vaccination and egg allergy. Can. Fam. Physician.

[12] Ramírez-Giraldo R.H., Giraldo-Avila P.A., Calle A.M., Santamaria L.C., Sánchez J. (2024). No yellow fever vaccine reactions in IgE-mediated egg allergic patients. Int. Arch. Allergy Immunol..

[13] García-Paba M.B., Aparicio C., Rodríguez M., Moreno S., García E. (2023). Frequency of allergic reactions in egg allergic patients after receiving the yellow fever vaccine. Allergol. Immunopathol..

[14] Tanos Lopes F.T., Maia de Castro Romanelli R., Isabela de Oliveira L., Abrantes M.M., Rocha W. (2023). Safe administration of yellow fever vaccine in patients with suspected egg allergy. J. Allergy Clin. Immunol. Glob..

[15] WHO Lower Doses of Yellow Fever Vaccine Could Be Used in Emergencies. Published 17 June 2016. https://www.who.int/news/item/17-06-2016--lower-doses-of-yellow-fever-vaccine-could-be-used-in-emergencies.

[16] Campi-Azevedo A.C., Reis L.R., Peruhype-Magalhães V., Coelho-dos-Reis J.G., Antonelli L.R., Fonseca C.T., Costa-Pereira C., Souza-Fagundes E.M., Costa-Rocha I.A.D., Mambrini J.V.D.M. (2019). Short-Lived Immunity After 17DD Yellow Fever Single Dose Indicates That Booster Vaccination May Be Required to Guarantee Protective Immunity in Children. Front. Immunol..

[17] Miyaji K.T., Avelino-Silva V.I., Simões M., Freire M.D.S., Medeiros C.R.D., Braga P.E., Neves M.A.A., Lopes M.H., Kallas E.G., Sartori A.M.C. (2017). Prevalence and titers of yellow fever virus neutralizing antibodies in previously vaccinated adults. Rev. Inst. Med. Trop. Sao Paulo.

[18] Saucha C.V.V., de Oliveira R.V.C., Maia M.L.S., Sousa E.S.S., de Oliveira P.M.N., Xavier J.R., de Castro T.D.M., Cruz R.L.D.S., Schwarcz W.D., Pereira R.C. (2026). A cohort study of the duration of immunity to yellow fever after 4 years of primary vaccination in children and adults. Trans. R. Soc. Trop. Med. Hyg..

[19] Ferrara P., La Vecchia A., Losa L., Mantovani L.G., Plana M., Agüero F. (2025). Is a second dose of yellow fever vaccine needed? A systematic review of humoral and cell-mediated immunity after revaccination. J. Travel Med..

